# 

**Published:** 1850-05

**Authors:** 


### CONTENTS:

### PART I.—ORIGINAL COMMUNICATIONS.

Davis' Medical History, - - - - - - - - - 3
ART. 1.—Malaria, and its Connection with the Existence and Character of Disease—By Samuel Thompson, M.D., - - - - - - 19
ART. 2.—Hemorrhoids with Scirrhous Rectum—By R. Willard, M.D., 43
ART. 3.—Observations on the use of Coffee as a cause of Disease—By Wm. Matthews, M.D., - - - - - - - - - 46
ART. 4.—Singular Case of Malformation—By C. A. Hathwell, M.D., - 50
ART. 5.—The Obstetrical Extractor—By John Evans, M.D., etc., - - 53
ART. 6.—A case of Uterine Hemorrhage—By Isaac E. Thayer, M.D., - 63
ART. 7.—Western Medical Society of the State of Wisconsin, - - 64

### PART II.—REVIEWS AND NOTICES OF NEW WORKS.

ART. 1.—Transactions of the American Medical Association, - - 65
ART. 2.—Principles of Human Physiology, with their Chief Applications to Pathology, Hygiene, and Forensic Medicine—By Wm. Carpenter, M.D., F.R.S., F.G.S., etc. - - - - - - - 73
ART. 3.—Diseases of Infants and Children—By Fleetwood Churchill, M.D. 75
ART. 4.—A Practical Treatise on the Diseases of Children—By D Fraucis Condie, M.D., etc. - - - - - - - - 76
ART. 5.—The Cholera in Cincinnati; or a connected view of the controversy between the Homœopathists and the Methodist Expositor—By Dr. S. A: Latta, - - - - - - - - - - 76
ART. 6.—A Systematic Treatise, etc., on the Principal Diseases of the Interior Valley of North America, as they appear in the Caucasian, African, and Esquimaux varieties of its population—By Daniel Drake, M.D, - 79
ART. 7.—Ranking's Abstract and Braithwaite's Retrospect, - - - 79

### PART III.—SELECTIONS.

ART. 1.—On the Treatment of External Hemorrhoids by Potassa Fusa—By Dr. F. C. Jones, - - - - - - - - - 80
ART. 2.—Treatment of Ulcers by means of Opium—By F. C. Skey, Esq., 80
ART 3.—Tannate of Quinine—By Dr. R. H. Thomas, - - - 81

### PART IV.—EDITORIAL.

ART. 1.—Free Medical Schools, - - - - - - - 82
ART. 2.—Illinois State Medical Convention - - - - - - 84
ART. 3.—Graduates in Rush Medical College—Session of 1849-50, - - 86
ART. 4.—Statistics of Medical Schools, - - - - - - 87
ART. 5.—Chicago, Medical Society, - - - - - - - 88
ART. 6.—Demonstrative Midwifery, - - - - - - 88
ART. 7.—Miscellaneous Medical Intelligence, - - - - - 89
ART. 8.—Northern Lancet—ART. 9.—Obituary, - - - - - 90

